# Considering the First Steps toward a Stable and Orderly Way of Bacterial Life

**DOI:** 10.1371/journal.pbio.0060180

**Published:** 2008-07-15

**Authors:** Karen Otto

## Abstract

The development of a short-time scale colloidal system introduces flow cytometry as a tool to investigate both physicochemical and molecular aspects of bacterial adhesion to solid surfaces.

Bacteria are small unicellular organisms who could well enjoy a bohemian life—moving independently wherever and whenever they want to and existing with no regard for conventional rules of behavior. In spite of this apparent freedom, most bacteria abandon their footloose lifestyle as soon as they come into contact with a surface. Irrespective of whether the surface is of biotic or abiotic origin, they clinch to it, forgoing independence in favor of settling down. Similar to animals that gather in flocks and people who live in societies, surface-attached microbes can form networks as multicellular communities called biofilms. Bacterial biofilms are heterogeneous structures of increasing complexity that consist of differently specialized cells enclosed in a self-produced polymeric matrix associated with the surface [1].

Depending on the setting and the composition of biofilms, they may have either beneficial or detrimental effects on our environment and health. One of the most serious concerns about biofilms is their high antibiotic tolerance, which makes the treatment of infections difficult and contributes to the spread of antibiotic resistance among pathogenic bacteria [2]. A high antibiotic tolerance in biofilm bacteria can partly be explained by a surface-induced change in gene expression, but how does this happen? What are the first critical steps towards an orderly life on a surface and how are these controlled?

These questions have puzzled scientists for a considerable amount of time, and still do. Attempts to solve these questions can be roughly divided into two major approaches: one focusing on physicochemical aspects of cell–surface interactions, the other aiming at elucidating the expression of adhesion-specific genes. When bacteria approach a surface they encounter an energy barrier, and a balance of repulsive and attractive forces determines whether adhesion occurs. Several theoretical models originally developed for colloidal particles have been used to describe this process, including the Derjaguin-Landau-Verwey-Overbeek (DLVO) theory [3], the thermodynamic approach [4], and an extended DLVO theory [5]. However, the predictions made for cell–surface interactions in terms of electrostatic interaction forces or interfacial energy have only limited success, because they do not take into account the heterogeneity of bacterial cell surfaces [6]. Bacterial cells not only have a variety of cell surface structures, they also sense and respond to changes in their environment by immediately adjusting their gene expression, which results in dynamic cell surface alterations.

Among surface-induced cellular changes, an altered expression of cell envelope components is of particular interest because it directly affects the mode of intimate cell–surface contact. To understand the genetic basis of the decisions bacteria make upon surface contact, many studies have focused either on the characterization of adhesion factors or on the isolation of biofilm-deficient mutants. During the past decade, a huge interest in biofilm research has resulted in amazing insights into how various cell surface structures affect the rate and extent of attachment, e.g., [7–10], how biofilms grow and develop in coordinated steps, e.g., [11–16], how the extracellular matrix is produced, e.g., [17,18], and how bacteria inside biofilms communicate via signal molecules, e.g., [17,19].

However, the very first steps that actually cause bacteria to stick to a surface and that are required to trigger reprogramming of gene expression are still not well understood. To a large extent, this lack of knowledge is due to a lack of appropriate methodology.

## Use of Flow Cytometry to Investigate the Initial Stage of Biofilm Formation

In this issue of *PLoS Biology*, Beloin and colleagues introduce a simple experimental approach that promises to shed new light on the very early events of the adhesion process [20]. They developed a micrometric colloidal adhesion assay that allows the study of the initial events of bacterial adhesion by flow cytometry.

Flow cytometry is a sensitive and efficient method used to characterize multiple physical parameters of suspended cells or small particles (0.2–150 micrometers in size) as they flow, one by one, in a narrow stream through a beam of light [21]. Detectors measure both forward light scattering (i.e., relative size) and side light scattering (i.e., internal complexity) as well as fluorescence emission for each cell that passes through the light source. The optical signals are collected by lenses, directed to the appropriate detectors and converted into digital signals, which are recorded and analyzed in real time [22]. The major advantage of this sensitive method is that it allows fast performance and a large throughput of analyzed cells.

While flow cytometry is a well-established tool for the characterization of eukaryotic cells and their interactions, it has apparently still not reached its full potential for microbiological applications [22]. Examples of studies range from analysis of the bacterial cell cycle, assessment of antibiotic susceptibility, and monitoring of microorganisms in environmental samples, to the determination of expression of intracellular or cell surface antigens [21].

Surprisingly few studies exist where flow cytometry was applied to study bacterial adhesion, and these mainly focus on aspects of adherence to epithelial mammal cells, e.g., [23–25]. To this end, bacteria were allowed to co-incubate with their host cells, which subsequently were collected, resuspended in buffer, and analyzed by flow cytometry. This assay did not only prove to be faster and more efficient than conventional adhesion assays, but also allowed for detection of weak bacterium–cell interactions [23]. Only one previous attempt has been made to use flow cytometry to study adhesion to solid surfaces [26]. In that study, in order to compare growth characteristics of attached cells with those of suspended cells, attached cells were removed from the surface by sonication prior to analysis. However, a procedure that interrupts direct cell–surface interactions is obviously not suitable for studies in which adhesion kinetics or dynamics are supposed to be the subject of investigation. Is it possible to use flow cytometry for the analysis of bacterial cells attached to solid surfaces without destroying their inherent characteristics?

## A Short–Time Scale Colloidal System

The current work by Beloin et al. [20] shows that it is. To avoid the practical problems connected with collecting attached cells from flat surfaces, the authors suggest a simple adhesion assay using colloidal particles with a diameter of 10 μm as adhesion substrate (Figure 1). An excess of bacterial cells, marked with green fluorescent protein (GFP), are mixed with fluorescently labeled colloidal particles, and aliquots are removed from this sample within seconds and at various time points for immediate analysis by flow cytometry. For data analysis, light scattering signals and two fluorescent signals are collected, one for bacteria and one for particles. In this way, it is possible to distinguish between colonized and uncolonized particles, as well as single bacteria and bacterial aggregates. Since the fluorescent intensity of bacteria is not affected by adhesion, the number of attached cells per particle could be calculated simply by dividing the particle fluorescence of one particle (averaged on the whole particle population) by the individual bacterial fluorescence (averaged on free cells passing the light source).

**Figure 1:**
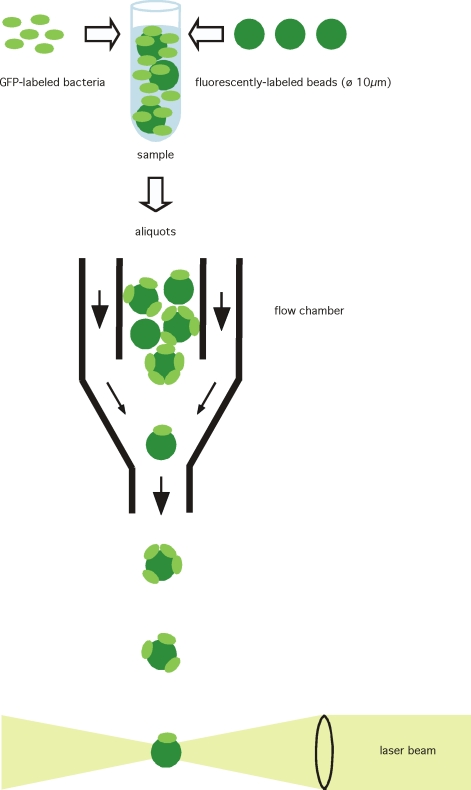
Schematic Presentation of the Micrometric Short–Time Scale Colloidal Assay GFP-labeled bacteria are mixed with fluorescently labeled particles at a ratio of 200 to one. The mixture is stirred by vortexing and first aliquots are removed within seconds for analysis by flow cytometry.

To exemplify the potential of flow cytometry for the analysis of quantitative as well as dynamic aspects of adhesion, the authors chose to study the influence of some well-characterized adhesion factors on the early adhesion process. All of these factors contributed to distinct adhesion kinetics.

For instance, curli are thin hairlike protein structures extruding from the cell surface and well known to mediate attachment to both living and abiotic surfaces [27]. As expected, fluorescence intensity measurements indicated that cells expressing curli bind to beads to a much higher degree than curli-deficient cells. More surprising is the finding that curli-dependent adhesion is a two-phase process. After a first adhesion plateau with three to four bacteria per particle has been reached, a sudden increase in adhesion occurs after about ten minutes, resulting in particles colonized with about 20 bacteria. Interestingly, this coincides with an increase in curli-mediated aggregation, observed to occur independently from attachment and as a concomitant increase in fluorescence intensity and forward light scattering.

The initially sparse colonization of particles led the authors to take a closer look at the particle surfaces. To investigate the origin of what they assumed to be a repulsion potential, they again used flow cytometry to determine the surface charge of the particles by measuring fluorescence of the cationic dye propidium iodide. On uncolonized cationic particles, propidium iodide did not bind, and no fluorescence signal was detected. However, as soon as bacteria colonized the surface, the fluorescence signal increased, indicating an immediate charge conversion on the surface.

It is well known that surfaces are conditioned with a film of proteins within seconds of exposure to biological fluids [28]. However, in this case it is striking that the bacteria-derived anionic molecule(s) appear to be actively secreted and accumulated, even on negatively charged surfaces. The immediate modification by the still unidentified molecule(s) is speculated to prime the surface contact of only a few cells by providing a limited number of links for the formation of adhesive bonds. Previously, secreted group II capsular polysaccharides have been identified as being involved in surface modification and inhibition of adhesion [29].

## Perspective

Using flow cytometry as a tool to explore initial cell–surface interactions will certainly provide us with new insights into the process of adhesion and biofilm formation. In addition to fluorescent markers that enable us to detect cells and particles or to identify the molecular composition of structures, fluorescent indicators could be used to monitor the physiological state of a cell in response to surface attachment. With a wide array of available fluorescent dyes and GFP variants at hand, the intricate relationships between physicochemical surface properties, cell surface structures, and surface-induced gene expression can be examined simultaneously and on a molecular level.

Although the possibility of analyzing single cells was not explored in this study, the potential exists. Many flow cytometers have the extended function of cell sorting. In these cytometers, the stream is broken up into drops that each contain one single cell [22]. Cells that exit the laser beam can be collected and analyzed, and the genes responsible for the phenotype in question identified. With the development of this assay, Beloin et al. give us a tool at hand that can be useful in the efficient search for adhesion mutants that have not yet been isolated in established screening assays.

## Abbreviations

DLVO: Derjaguin-Landau-Verwey-Overbeek
GFP: green fluorescent protein
